# Serum PM20D1 levels in patients with idiopathic pulmonary arterial hypertension and its clinical significance

**DOI:** 10.1186/s12872-024-03855-6

**Published:** 2024-04-13

**Authors:** Lin Wang, Jiaxiang Liu, Liufang Zhou, Qingmei Fu

**Affiliations:** 1 Department of Respiratory and Critical Care Medicine, Zhuzhou Hospital Affiliated to Xiangya School of Medicine, Central South University, Zhuzhou, 412000 Hunan Province P.R. China; 2 Department of Cardiology, Zhuzhou Hospital Affiliated to Xiangya School of Medicine, Central South University, Zhuzhou, 412000 Hunan Province P.R. China; 3 Department of Anesthesiology, Zhuzhou Hospital Affiliated to Xiangya School of Medicine, Central South University, Zhuzhou, 412000 Hunan Province P.R. China; 4 Department of Ultrasound, Zhuzhou Hospital Affiliated to Xiangya School of Medicine, Central South University, No.116, Changjiang South Road, Tianyuan District, Zhuzhou City, 412000 Hunan Province P.R. China

**Keywords:** Idiopathic pulmonary arterial hypertension, PM20D1, Lipid, Prognosis

## Abstract

**Objective:**

This study aimed to investigate the serum levels of Peptidase M20 domain containing 1 (PM20D1) in idiopathic pulmonary arterial hypertension (IPAH) patients and examine its association with lipid metabolism, echocardiography, and hemodynamic parameters.

**Methods:**

This prospective observational research enrolled 103 IPAH patients from January 2018 to January 2022. Enzyme-linked immunosorbent assay (ELISA) was used to measure the serum PM20D1 levels in all patients before treatment within 24 h of admission. Demographic data, echocardiography, hemodynamic parameters and serum biomarkers were also collected.

**Results:**

The IPAH patients in the deceased group had significantly elevated age, right atrial (RA), mean pulmonary arterial pressure (mPAP), mean right atrial pressure (mRAP), pulmonary capillary wedge pressure (PCWP), pulmonary vascular resistance (PVR) and significantly decreased 6 min walking distance (6MWD) and tricuspid annulus peak systolic velocity (TASPV). IPAH patients showed significant decreases in serum PM20D1, low-density lipoprotein cholesterol (LDL-C), and albumin (ALB). Additionally, PM20D1 was negatively correlated with RA, NT-proBNP and positively correlated with PVR, ALB, 6MWD, and TAPSV. Moreover, PM20D1 has the potential as a biomarker for predicting IPAH patients’ prognosis. Finally, logistic regression analysis indicated that PM20D1, ALB, NT-proBNP, PVR, TASPV, RA and 6MWD were identified as risk factors for mortality in IPAH patients.

**Conclusion:**

Our findings indicated that the serum levels of PM20D1 were significantly decreased in IPAH patients with poor prognosis. Moreover, PM20D1 was identified as a risk factor associated with mortality in IPAH patients.

**Supplementary Information:**

The online version contains supplementary material available at 10.1186/s12872-024-03855-6.

## Introduction

Pulmonary Arterial Hypertension (PAH) is a serious pulmonary vascular disease characterized by elevated resting mean pulmonary arterial pressure (mPAP) [1]. PAH affects the function of pulmonary arteries and pulmonary capillaries, leading to ineffective blood flow [2, 3]. PAH is a rare condition, with reported prevalence ranging from 15 to 50 cases per million individuals in the United States and Europe [4]. Idiopathic Pulmonary Arterial Hypertension (IPAH) is a subtype of PAH that can cause pulmonary artery constriction and proliferation, resulting in increased pulmonary vascular resistance, right heart failure, and ultimately death [5, 6]. Therefore, early prediction of patient prognosis is crucial and can guide more aggressive treatment decisions.

Research has found an association between right ventricular dysfunction in PAH patients and lipid metabolism [7, 8]. This suggests the presence of lipid metabolism abnormalities in PAH patients. Peptidase M20 domain containing 1 (PM20D1) is an enzyme found in adipocytes that is secreted and has recently been discovered and highly expressed in adipocytes that contain uncoupling protein-1 (UCP1) [9]. PM20D1 is a protein associated with lipid metabolism. Upregulation of PM20D1 expression activates the phosphatidylinositol 3-kinase (PI3K)/Akt signaling pathway, improving lipid breakdown in adipocytes [10]. In preliminary observations from our earlier research, we identified abnormal PM20D1 expression in PAH patients (not published data). Furthermore, decreased expression of PM20D1 has been observed in cardiovascular diseases such as atherosclerosis and gestational diabetes, and lower PM20D1 levels are associated with increased secretion of inflammatory factors, impaired lipid metabolism, and worse prognosis in patients [11, 12]. However, currently, there are no clinical studies investigating the relationship between PM20D1 and lipid metabolism factors in IPAH patients, as well as its impact on patient prognosis.

In this prospective observational study, our objective was to investigate the serum levels of PM20D1 in IPAH patients and examine its association with lipid metabolism, echocardiography, and hemodynamic parameters. The findings from this study could elucidate the clinical relevance of PM20D1 in IPAH patients, and also present novel avenues for future research in IPAH treatment.

## Methods

### Participants

This prospective observational research enrolled 103 IPAH patients who came to our hospitals for treatment from January 2018 to January 2022. All patients are older than 18 years and were diagnosed with IPAH according to the 2022 ESC/ERS Guidelines for the diagnosis and treatment of pulmonary hypertension [13]. The treatment of all study participants was conducted based on the recommendations outlined in this guideline. Exclusion criteria include: (1) secondary pulmonary arterial hypertension caused by left heart disease, lung disease, or pulmonary embolism; (2) patients with congenital heart disease, cardiomyopathy, or severe arrhythmias; (3) patients with severe infection, liver or kidney dysfunction, or malignant tumors; (4) patients undergoing nutritional therapy, anti-inflammatory or immunotherapy; (5) Patients with recent acute myocardial infarction or pulmonary embolism or other acute events that may acutely increase troponin levels. In addition, we also collected serum samples from 100 healthy volunteers who came to our hospital for physical examination as control. All healthy volunteers were comparable to the patients in terms of age and gender, and they had no organic heart disease or lung disease. Additionally, their liver and kidney function, blood glucose levels, and chest X-ray results were all within the normal range. All study subjects were not treated with ongoing hypolipidemic therapy at the time of enrollment. This research received ethical approval from the Ethics Committees of our hospital. All enrolled individuals agreed to take part in this study and signed the consent informed forms.

### Echocardiography and right heart catheterization

We used Vivid 7 ultrasound platform (GE Healthcare, Milwaukee, Wisconsin) to collect ultrasound echocardiography data from patients, including right atrial (RA) measurements, right ventricle (RV) measurements, pulmonary artery width (PAW), pulmonary artery velocity (PAV), tricuspid annular plane systolic excursion (TAPSE), tricuspid annulus peak systolic velocity (TASPV), and pulmonary arterial systolic pressure (PASP). Additionally, all patients underwent right heart catheterization before treatment to obtain hemodynamic parameters, including mean pulmonary arterial pressure (mPAP), pulmonary capillary wedge pressure (PCWP), pulmonary vascular resistance (PVR), pulmonary vascular resistance index (PVRI), and mean right atrial pressure (mRAP).

### Blood sampling measurement

Enzyme-linked immunosorbent assay (ELISA) was used to measure the serum PM20D1 levels in all patients before treatment within 24 h of admission. Briefly, fasting venous blood samples (5 mL) were collected from all study participants. Subsequently, the samples were centrifuged at 2000 g for 15 min and analyzed according to the instructions provided by the commercially available assay kit (MBS280518, MyBioSource, USA).

### Observation indicators

The observed variables include age, BMI, gender, WHO-FC, and 6 min walking distanc (6MWD) for all study participants. Additionally, we utilized a fully automated biochemical analyzer (Hitachi 7600, Hitachi Ltd., Japan) to perform a complete blood panel analysis, recording levels of low-density lipoprotein cholesterol (LDL-C), high-density lipoprotein cholesterol (HDL-C), triglycerides (TG), total cholesterol (TC), serum albumin (ALB), N terminal pro B type natriuretic peptide (NT-ProBNP) and cardiac troponin T (cTnT) in patients. In addition, all patients were followed up for 540 days, and during the follow-up period, the overall survival (OS) of all patients were recorded.

### Statistical analysis

All data used SPSS 26.0 for analysis. The comparison between two groups with a normal distribution was conducted using the Mann-Whitney test. For the comparison between two groups with a non-normal distribution, the Student’s *t*-test was employed. One-way analysis of variance (ANOVA) followed by Tukey’s post hoc test and Bonferroni correction was used for comparison among three groups. The Chi-square test was used for comparing ratios. Spearman’s rank correlation was used for correlation analysis. The role of serum PM20D1 in the prediction of patient prognosis was analyzed using ROC curve analysis. Kaplan-Meier (K-M) curve analysis was utilized to compare overall survival (OS) among patients. Multivariable logistic regression analysis was performed to identify risk factors associated with poor prognosis in IPAH patients. *P* < 0.05 regarded a significant difference.

## Results

### Clinical characteristics of all participants

This prospective observational study included 103 patients with IPAH who received treatment at our hospital. All patients were followed up for 540 days and were divided into a survival group (*n* = 72) and a deceased group (*n* = 31) based on mortality status. 38.7% of female patients deceased during the follow-up period. When comparing demographic data, echocardiography, and hemodynamic parameters between the two groups, we found that compared to the survival group, patients in the deceased group had significantly elevated age, RA measurements and significantly decreased 6MWD and TAPSV (Table 1, *P* < 0.05). Additionally, hemodynamic parameters showed that the mPAP, PCWP and PVR were remarkably elevated in deceased group compared to the survival group (*P* < 0.05).

**Table 1:** Demographic and clinical data of all subjects

Variable	Deceased group, *n* = 31	Survival group, *n* = 72	p
Age, y	52.87 ± 9.92	46.97 ± 11.86	0.017
Sex, female (%)	12 (38.7)	27 (37.5)	0.885
BMI	24.14 (20.30-28.36)	24.75 (19.95–28.10)	0.980
WHO-FC			
I-II, n (%)	0 (0%)	23 (31.9)	
III-IV, n (%)	31 (100%)	49 (68.1)	
6MWD (m)	230.70 ± 53.56	333.95 ± 35.17	< 0.001
RA (mm)	51.47 (43.54–61.88)	44.99 (34.80-53.36)	< 0.001
RV (mm)	56.97 ± 4.30	58.57 ± 4.24	0.082
PAW (mm)	33.39 ± 2.96	33.23 ± 2.99	0.803
PAV (m/s)	0.67 ± 0.08	0.65 ± 0.86	0.424
TASPE (mm)	14.93 ± 2.43	14.47 ± 2.73	0.423
TASPV (cm/s)	7.41 (4.97–9.59)	9.66 (6.94–11.56)	< 0.001
PASP (mmHg)	86.67 ± 4.57	85.67 ± 4.96	0.340
MPAP (mmHg)	62.44 ± 5.12	60.12 ± 5.30	0.043
PCWP (mmHg)	11.47 (8.32–14.94)	9.43 (7.52–11.72)	< 0.001
PVR (wood)	20.95 ± 2.45	17.56 ± 2.38	< 0.001
PVRI (wood/m^2^)	2.67 ± 0.36	2.73 ± 0.47	0.554
MRAP (mmHg)	7.56 ± 1.76	7.69 ± 1.55	0.710

BMI – body mass index; 6MWD – 6 min walking distance; RA – right atrial; RV – right ventricle; PAW – pulmonary artery width; PAV – pulmonary artery velocity; TAPSE – tricuspid annular plane systolic excursion; TASPV – tricuspid annular plane systolic excursion; PASP – pulmonary arterial systolic pressure; mPAP – mean pulmonary arterial pressure; PCWP – pulmonary capillary wedge pressure; PVR – pulmonary vascular resistance; PVRI – pulmonary vascular resistance index; mRAP – mean right atrial pressure. Continuous data presented non-normal distribution were expressed by median (minimum to maximum) and analyzed by Mann-Whitney U test. Continuous data presented normal distribution were expressed by mean ± SD and analyzed by Student’s t test. Chi square test was used for rates.

### PM20D1 levels and other serum biomarkers in IPAH patients

**Fig. 1:**
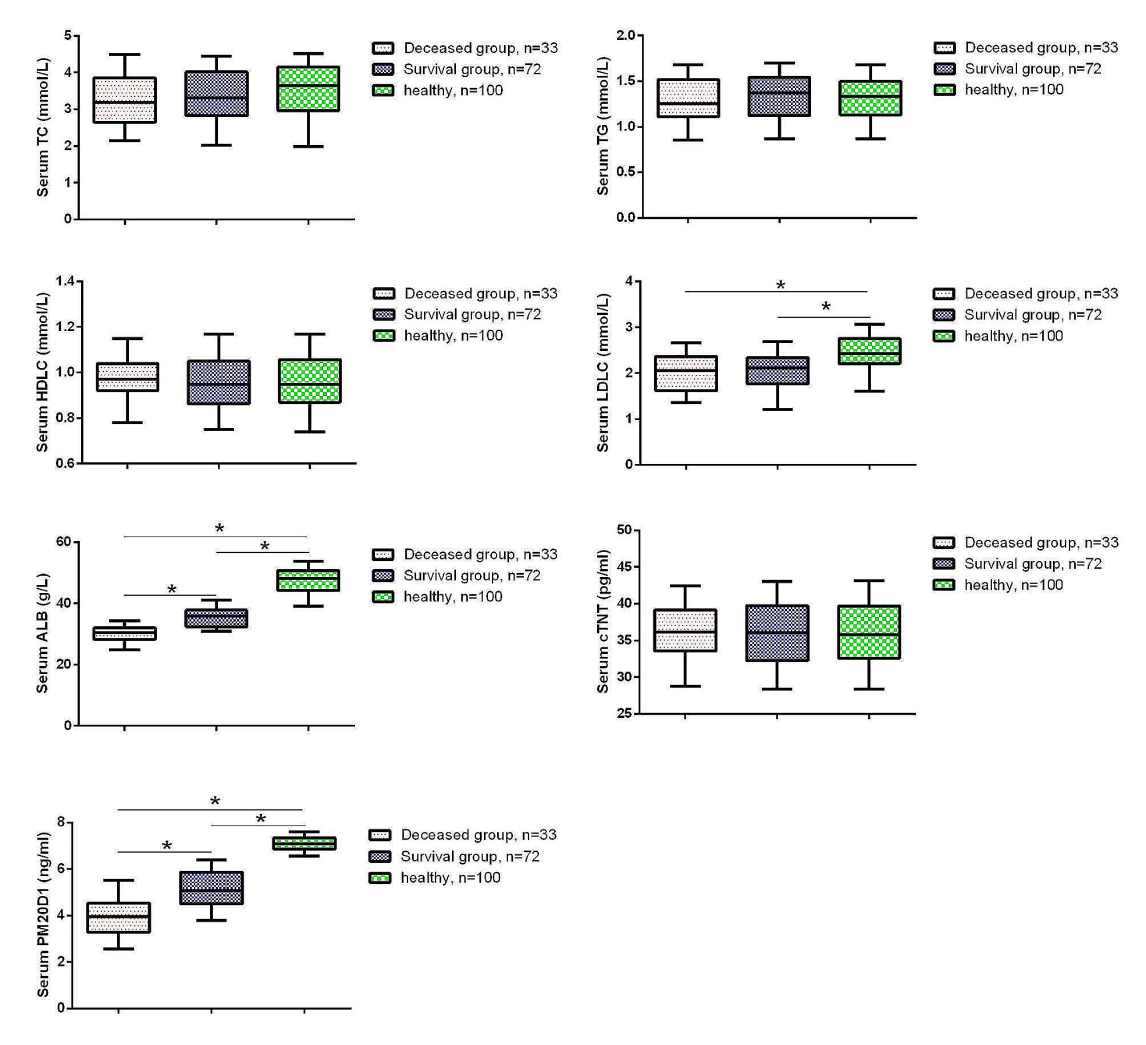
The expression of serum PM20D1, TC, TG, HDL-C, LDL-C, ALB and cTNT. *P < 0.05. One-way analysis of variance (ANOVA) followed by Tukey’s post hoc test and Bonferroni correction was used for comparison among three groups

Subsequently, we also measured the levels of serum biomarkers PM20D1, TC, TG, HDL-C, LDL-C, ALB, and cTnT in all IPAH patients and healthy volunteers. Compared to healthy volunteers, IPAH patients showed significant decreases in serum PM20D1, LDL-C, and ALB (Fig. 1, *P* < 0.05). Additionally, compared to the survival group, the deceased group of IPAH patients had significantly lower levels of PM20D1 and ALB in serum (*P* < 0.05).

### Correlation between serum PM20D1 levels and clinical outcome in IPAH patients

We subsequently conducted Spearman rank correlation analysis to investigate the association between PM20D1 levels and clinical outcomes in IPAH patients. As presented in Table 2, no significant correlation was observed between PM20D1 levels and age, BMI, TC, TG, HDL-C, cTNT, RV, PAW, PAV, TASPE, PASP, MPAP, PVR, PVRI and MRAP. However, a positive correlation was found between PM20D1 andPVR, ALB, 6MWD and TAPSV (*p* < 0.05). Additionally, PM20D1 was negatively correlated with RA and NT-proBNP (*p* < 0.05). These results suggested that PM20D1 might be linked to clinical outcomes in IPAH patients.

**Table 2:** Correlation between serum PM20D1 levels and clinical outcome

Variable	PM20D1	
	Spearman’s correlation	P
Age	0.03.6	0.721
BMI	0.055	0.578
6MWD	0.417	< 0.001
RA	-0.266	0.007
RV	-0.036	0.720
PAW	0.017	0.868
PAV	0.026	0.798
TASPE	-0.106	0.286
TASPV	0.297	0.002
PASP	-0.089	0.371
MPAP	-0.122	0.220
PCWP	-0.179	0.070
PVR	-0.194	0.049
PVRI	0.113	0.257
MRAP	0.024	0.806
TC	0.098	0.324
TG	-0.017	0.863
HDL-C	-0.149	0.134
LDL-C	-0.085	0.393
ALB	0.365	< 0.001
NT-proBNP	-0.482	< 0.001
cTNT	0.109	0.273

BMI – body mass index; 6MWD – 6 min walking distance; RA – right atrial; RV – right ventricle; PAW – pulmonary artery width; PAV – pulmonary artery velocity; TAPSE – tricuspid annular plane systolic excursion; TASPV – tricuspid annular plane systolic excursion; PASP – pulmonary arterial systolic pressure; mPAP – mean pulmonary arterial pressure; PCWP – pulmonary capillary wedge pressure; PVR – pulmonary vascular resistance; PVRI – pulmonary vascular resistance index; mRAP – mean right atrial pressure; LDL-C – low-density lipoprotein cholesterol; HDL-C – high-density lipoprotein cholesterol; TG – triglycerides; TC – total cholesterol; ALB – albumin; NT-ProBNP - N terminal pro B type natriuretic peptide; cTnT – cardiac troponin T.

### K-M curve analysis of serum PM20D1 in IPAH patients

**Fig. 2:**
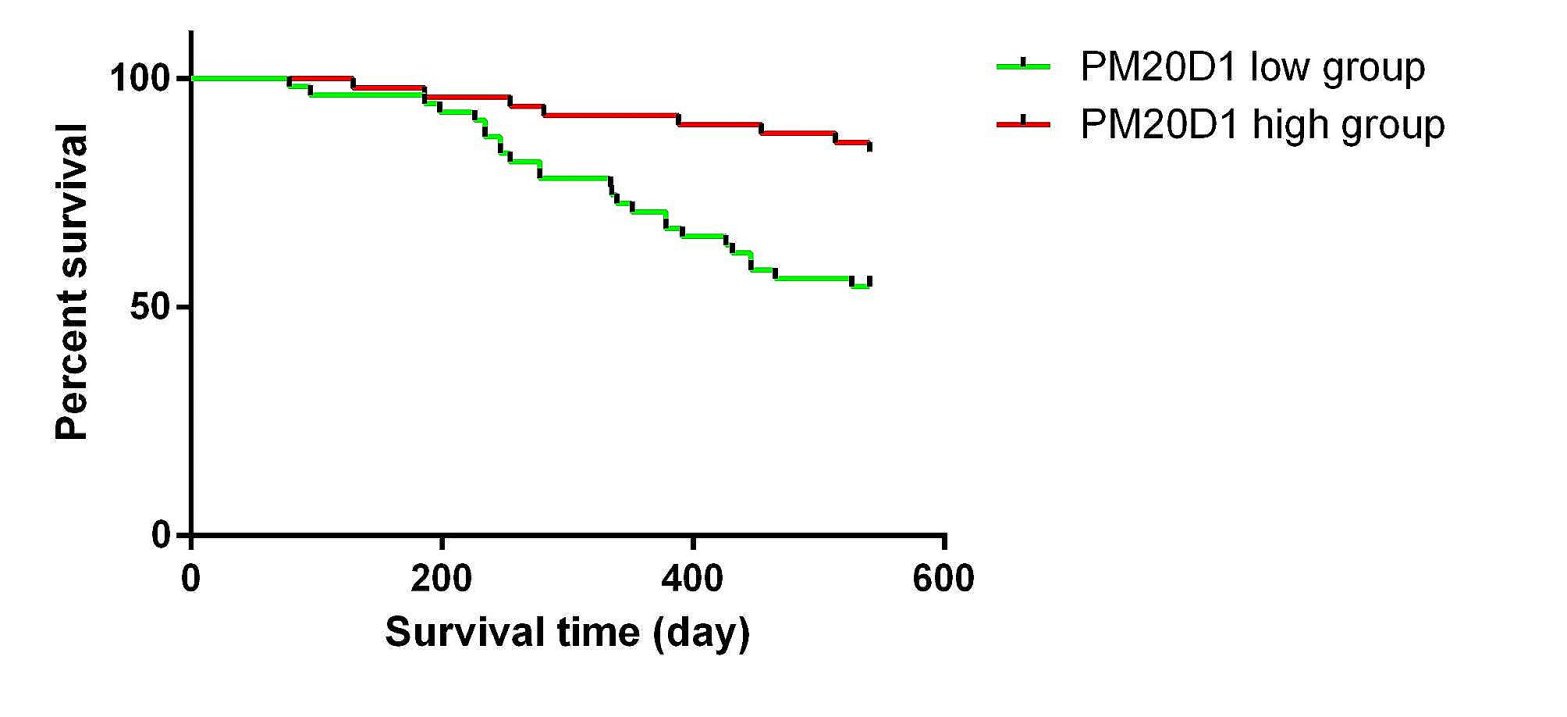
K-M curve for patients with high/low PM20D1.

We investigated the 540 days survival and relapse condition in two groups of IPAH patients categorized based on high/low serum PM20D1 levels (mean value 4.76 ng/ml) using K-M curve analysis. The median follow-up time for all patients was 540 (78–540) days, with a median follow-up time of 540 (78–540) days for the low PM20D1 levels group and a median follow-up time of 540 (129–540) days for the high PM20D1 levels group. The results revealed that the low-level group exhibited a shorter 540 days survival time (Fig. 2).

### Predictive value of PM20D1 for survival in IPAH patients

**Fig. 3:**
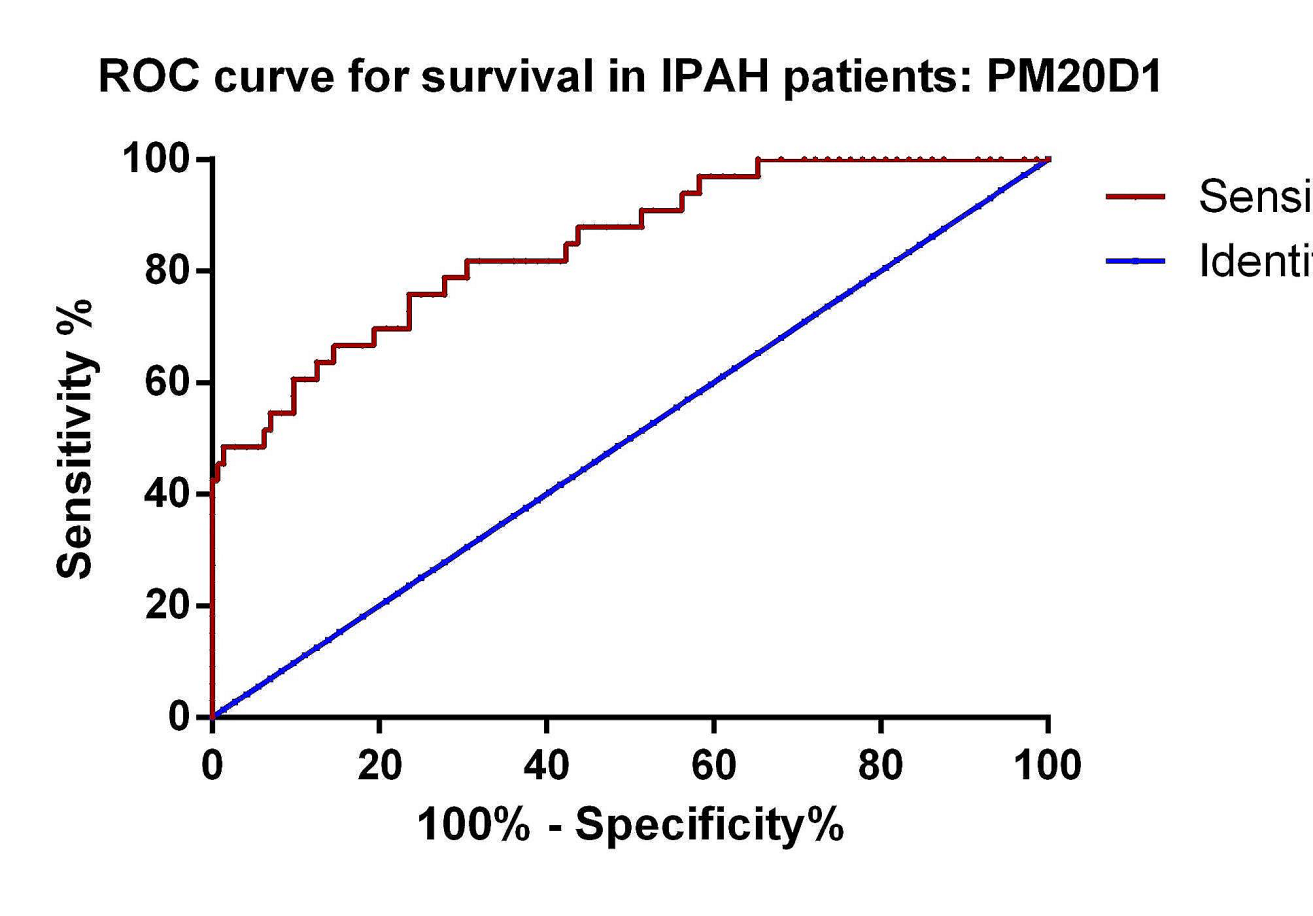
ROC curve of diagnostic value of PM20D1 for patients’ mortality

To assess the prognostic value of PM20D1 in IPAH patients, we constructed ROC curves. The results indicated that PM20D1 has potential as a biomarker for predicting IPAH patients’ prognosis (Fig. 3). The AUC of PM20D1 in predicting survival in IPAH patients was 0.845, 95% confidence intervals 0.763–0.927, with a cutoff value of 4.48 ng/ml, sensitivity of 76.4%, and specificity of 74.2%.

### Identification of risk factors for mortality in IPAH patients using logistic regression analysis

To determine the risk factors for mortality in IPAH patients, we performed multivariable logistic regression analysis using the enter method. In this analysis, we established three models, including Model 1 for demographic data, Model 2 for echocardiography and hemodynamic parameters, and Model 3 for serum biomarkers. The results indicated that PM20D1, ALB, NT-proBNP, PVR, TASPV, RA and 6MWD were identified as risk factors for mortality in IPAH patients (Table 3).

**Table 3:** Logistic regression of risk factors for death in IPAH patients

Variables	Wald	Odds ratio	95% CI	P
Age	0.649	0.972	0.906–1.042	0.421
BMI	0.991	2.281	0.450-11.577	0.320
Sex	0.944	0.853	0.619–1.176	0.331
6MWD	22.068	1.055	1.031–1.078	< 0.001
RA	7.437	0.531	0.337–0.837	0.006
RV	2.930	1.481	0.945–2.321	0.087
PAW	0.022	1.040	0.613–1.765	0.883
PAV	1.138	0.001	0.001-2106.658	0.286
TASPE	1.347	0.763	0.483–1.205	0.246
TASPV	6.226	12.416	1.717–89.801	0.013
PASP	0.137	1.059	0.783–1.431	0.711
MPAP	0.944	0.807	0.524–1.244	0.331
PCWP	3.686	0.311	0.094–1.025	0.055
PVR	4.809	0.551	0.323–0.939	0.028
PVRI	0.685	0.211	0.005–8.407	0.408
MRAP	1.017	0.483	0.118–1.986	0.313
TC	2.391	3.193	0.733–13.913	0.122
TG	0.001	1.004	0.032–31.602	0.998
HDL-C	3.308	7574.119	0.500-1.148E8	0.069
LDL-C	2.092	6.483	0.515–81.633	0.148
ALB	8.220	3.145	1.437–6.882	0.004
NT-proBNP	5.220	0.998	0.997-1.000	0.022
cTNT	2.401	0.795	0.595–1.063	0.121
PM20D1	8.958	20.579	2.840-149.112	0.003

BMI – body mass index; 6MWD – 6 min walking distance; RA – right atrial; RV – right ventricle; PAW – pulmonary artery width; PAV – pulmonary artery velocity; TAPSE – tricuspid annular plane systolic excursion; TASPV – tricuspid annular plane systolic excursion; PASP – pulmonary arterial systolic pressure; mPAP – mean pulmonary arterial pressure; PCWP – pulmonary capillary wedge pressure; PVR – pulmonary vascular resistance; PVRI – pulmonary vascular resistance index; mRAP – mean right atrial pressure; LDL-C – low-density lipoprotein cholesterol; HDL-C – high-density lipoprotein cholesterol; TG – triglycerides; TC – total cholesterol; ALB – albumin; NT-ProBNP - N terminal pro B type natriuretic peptide; cTnT – cardiac troponin T; PM20D1 – Peptidase M20 domain containing 1.

## Discussion

The prognosis of IPAH patients is poor, with a reported 3-year survival rate of approximately 80% ^14 15^. Early identification of biomarkers that reflect disease severity and prognosis in IPAH patients is of significant clinical value. Here, our results showed a significant decrease in serum PM20D1 levels in deceased IPAH patients. Furthermore, we identified serum PM20D1 as a potential predictor of survival in IPAH patients. This finding suggested that PM20D1 might play a crucial role in the pathophysiology of IPAH and could serve as a potential prognostic marker.

Previous studies have explored risk factors for mortality in IPAH. A study in Australia and New Zealand suggested that male gender and lower exercise capacity predict mortality in patients with IPAH [14]. Additionally, another research found that right heart reverse remodeling (RHRR) after one year of treatment is an independent predictor of prognosis in IPAH [15]. Furthermore, parameters such as 6MWD [16] and hemodynamic (mPAP, mRAP, PVR, and PCWP) [17] are significantly associated with mortality in IPAH. These findings are consistent with the results of our study, where we observed that deceased IPAH patients had higher age, mPAP, mRAP, PVR, PCWP, and lower 6MWD and TAPSE. Interestingly, we did not find an association between gender and mortality in IPAH patients, which may be related to differences in study sites and population ethnicity. Additionally, multivariable logistic regression analysis in our study showed that 6MWD and mPAP were risk factors for mortality in IPAH patients.

In recent years, researchers have started to focus on the potential application of serum biomarkers in predicting prognosis in PAH. Xu et al. demonstrated that baseline serum levels of direct bilirubin (DBIL) were significantly higher in non-survivors compared to survivors, and multivariate analysis showed that baseline DBIL was an independent risk factor for mortality in IPAH [18]. Zhang et al. confirmed that superoxide dismutase (SOD) activity in IPAH patients was significantly lower than in healthy individuals, and reduced extracellular SOD (Ec-SOD) activity increased the risk of death in patients [19]. Jonas et al. found that LDL-C levels were lower while the ratio of triglycerides to high-density lipoprotein cholesterol (TG/HDL-C) was higher in IPAH patients compared to the general population [20]. Consistent with these findings, our results also showed a decrease in LDL-C levels in IPAH patients compared to healthy volunteers.

Identifying clinically relevant prognostic factors to determine treatment goals is of paramount importance for improving long-term survival outcomes in IPAH patients. Matsubara et al. demonstrated that current treatment goals were insufficient to significantly enhance long-term survival rates in patients with IPAH. It has been observed that single-drug therapy is incapable of achieving a substantial reduction in afterload, thereby necessitating combination therapy involving multiple treatment modalities [21]. Therefore, further exploration of novel treatment targets for IPAH patients is deemed necessary. The effectiveness of PM20D1 targeting in inhibiting lipid metabolism and promoting lipid accumulation [22]. PM20D1 interacts with miR-324-5p to inhibit C2C12 cell differentiation and promote intramuscular lipid accumulation [23]. In our study, we measured serum levels of PM20D1 and lipid metabolism factors in IPAH patients, and found that deceased IPAH patients had lower levels of PM20D1 and ALB compared to surviving IPAH patients. This may be related to the protective effects of obesity in PAH [14]. Furthermore, correlation analysis also showed that PM20D1 was positively correlated not only with lipid metabolism factors but also with ALB. LDL-C is an important component of lipid metabolism, and theoretically, we expected a negative correlation between LDL-C and PM20D1. Additionally, it is also possible that higher levels of LDL-C may be associated with lower mortality risk. Some studies have reported lower mortality rates in severely ill patients with obesity, suggesting that higher adiposity may be beneficial for survival in critically ill patients at risk of death [24, 25]. However, these are speculative findings, and more data are needed to confirm these observations. Moreover, Logistic regression analysis also demonstrated that lower serum levels of PM20D1 and ALB were risk factors for mortality in IPAH patients. This is consistent with the findings of David et al. [26], who found a correlation between lower serum ALB concentrations and higher mortality rates in PAH patients. The downregulation of PM20D1 may also reflect differences in patients’ nutritional status [27], leading to adverse prognosis.

In clinical studies, the reduction of PM20D1 has been associated with clinical outcomes and pregnancy outcomes in gestational diabetes mellitus (GDM) patients [12]. Additionally, serum levels of PM20D1 have been found to decrease in patients with carotid atherosclerosis and are correlated with the severity of carotid atherosclerosis, plaque stability, and lipid metabolism levels [11]. This suggests that in future research, PM20D1 may have the potential for detecting other cardiovascular diseases. However, there are no further clinical studies on PM20D1. To our knowledge, we have found for the first time that serum PM20D1 levels are associated with lipid metabolism and prognosis in patients with IPAH. The correlation between PM20D1 and lipid metabolism factors in IPAH raises the possibility of targeting PM20D1 as a therapeutic strategy. By modulating the expression or activity of PM20D1, it may be possible to restore normal lipid metabolism and improve the prognosis of IPAH patients. This also indicates the potential clinical applicability of PM20D1. Future studies should explore the regulatory mechanisms of PM20D1 and its role in lipid metabolism in IPAH, as well as investigate the therapeutic potential of targeting PM20D1 in the treatment of IPAH.

Certain limitations to our study should be acknowledged. Firstly, the sample size was relatively small, which may limit the generalizability of our findings. Secondly, we did not measure changes in serum PM20D1 levels of all patients after treatment. Thirdly, our analysis only assessed a limited number of serum biomarkers, which may have excluded other potentially relevant variables. Lastly, further in-depth research is needed to elucidate the molecular mechanisms by which PM20D1 is involved in the development of IPAH.

## Conclusion

Our findings indicated that the serum levels of PM20D1 were significantly decreased in IPAH patients with poor prognosis. Moreover, PM20D1 was identified as a risk factor associated with death in IPAH patients. Further investigation into the role of PM20D1 in IPAH might provide new targets and a comprehensive approach to treatment in IPAH patients.

### Electronic supplementary material

Below is the link to the electronic supplementary material.

Supplementary Material 1

## Data Availability

The data underlying this article will be shared on reasonable request to the corresponding author.

## References

[1] Hassoun PM (2021). Pulmonary arterial hypertension. N Engl J Med Dec.

[2] Coons JC, Pogue K, Kolodziej AR, Hirsch GA, George MP (2019). Pulmonary arterial hypertension: a pharmacotherapeutic update. Curr Cardiol Rep Nov.

[3] Beshay S, Sahay S, Humbert M. Evaluation and management of pulmonary arterial hypertension. Respir Med. Sep 2020;171:106099. 10.1016/j.rmed.2020.106099. 10.1016/j.rmed.2020.106099 32829182

[4] Levine DJ (2021). Pulmonary arterial hypertension: updates in epidemiology and evaluation of patients. Am J Manag Care Mar.

[5] Swiston JR, Johnson SR, Granton JT (2010). Factors that prognosticate mortality in idiopathic pulmonary arterial hypertension: a systematic review of the literature. Respir Med Nov.

[6] Vazquez ZGS, Klinger JR (2020). Guidelines for the treatment of pulmonary arterial hypertension. Lung Aug.

[7] Agrawal V, Lahm T, Hansmann G, Hemnes AR (2020). Molecular mechanisms of right ventricular dysfunction in pulmonary arterial hypertension: focus on the coronary vasculature, sex hormones, and glucose/lipid metabolism. Cardiovasc Diagn Ther Oct.

[8] Talati M, Hemnes A (2015). Fatty acid metabolism in pulmonary arterial hypertension: role in right ventricular dysfunction and hypertrophy. Pulm Circ Jun.

[9] Sanchez-Mut JV, Heyn H, Silva BA, Dixsaut L, Garcia-Esparcia P, Vidal E, Sayols S, Glauser L, Monteagudo-Sánchez A, Perez-Tur J (2018). PM20D1 is a quantitative trait locus associated with Alzheimer’s disease. Nat Med.

[10] Li M, Gao S, Kang M, Zhang X, Lan P, Wu X, Yan X, Dang H, Zheng J (2023). Quercitrin alleviates lipid metabolism disorder in polycystic ovary syndrome-insulin resistance by upregulating PM20D1 in the PI3K/Akt pathway. Phytomedicine Aug.

[11] Huang X, He P, Wu L (2022). Clinical significance of peptidase M20 domain containing 1 ii patients with carotid atherosclerosis. Arquivos brasileiros de cardiologia.

[12] Hou J, Chen X, Xia J, Zhang L. Down-regulation of PM20D1 is associated with clinical outcomes and prognosis of pregnant patients with diabetes mellitus. Archives Med Sci. 2022. 10.5114/aoms/136284 PMC10696964 38058717

[13] Humbert M, Kovacs G, Hoeper MM, Badagliacca R, Berger RMF, Brida M, Carlsen J, Coats AJS, Escribano-Subias P, Ferrari P, Ferreira DS, Ghofrani HA, Giannakoulas G, Kiely DG, Mayer E, Meszaros G, Nagavci B, Olsson KM, Pepke-Zaba J, Quint JK, Rådegran G, Simonneau G, Sitbon O, Tonia T, Toshner M, Vachiery JL, Vonk Noordegraaf A, Delcroix M, Rosenkranz S (2022). 2022 ESC/ERS guidelines for the diagnosis and treatment of pulmonary hypertension. Eur Heart J Oct.

[14] Strange G, Lau EM, Giannoulatou E, Corrigan C, Kotlyar E, Kermeen F, Williams T, Celermajer DS, Dwyer N, Whitford H, Wrobel JP, Feenstra J, Lavender M, Whyte K, Collins N, Steele P, Proudman S, Thakkar V, Keating D, Keogh A (2018). Survival of idiopathic pulmonary arterial hypertension patients in the modern era in Australia and New Zealand. Heart Lung Circ Nov.

[15] Badagliacca R, Poscia R, Pezzuto B, Papa S, Reali M, Pesce F, Manzi G, Gianfrilli D, Ciciarello F, Sciomer S, Biondi-Zoccai G, Torre R, Fedele F, Vizza CD. Prognostic relevance of right heart reverse remodeling in idiopathic pulmonary arterial hypertension. J Heart Lung Transpl Oct. 2017;2. 10.1016/j.healun.2017.09.026. 10.1016/j.healun.2017.09.026 29107544

[16] Zhang Y, Chen Y, Yao H, Lie Z, Chen G, Tan H, Zhou Y (2019). Elevated serum circ_0068481 levels as a potential diagnostic and prognostic indicator in idiopathic pulmonary arterial hypertension. Pulm Circ Oct-Dec.

[17] Hu EC, He JG, Liu ZH, Ni XH, Zheng YG, Gu Q, Zhao ZH, Xiong CM (2015). High levels of serum lactate dehydrogenase correlate with the severity and mortality of idiopathic pulmonary arterial hypertension. Exp Ther Med Jun.

[18] Xu XQ, Lv ZC, Liu QQ, Zhao QH, Wu Y, Sun K, Jiang X, Wang L, Peng FH, Jing ZC (2017). Direct bilirubin: a new risk factor of adverse outcome in idiopathic pulmonary arterial hypertension. Int J Cardiol Feb 1.

[19] Zhang R, Wang L, Zhao QH, Jiang R, Gong SG, Jiang X, Xu XQ, He YY, Li Y, Jing ZC (2020). Alteration of Extracellular Superoxide dismutase in idiopathic pulmonary arterial hypertension. Front Med (Lausanne).

[20] Jonas K, Waligóra M, Magoń W, Zdrojewski T, Stokwiszewski J, Płazak W, Podolec P, Kopeć G (2019). Prognostic role of traditional cardiovascular risk factors in patients with idiopathic pulmonary arterial hypertension. Arch Med Sci Oct.

[21] Matsubara H, Ogawa A (2014). Treatment of idiopathic/hereditary pulmonary arterial hypertension. J Cardiol Oct.

[22] Li D, Liu Y, Gao W, Han J, Yuan R, Zhang M, Pang W (2019). Inhibition of mir-324-5p increases PM20D1-mediated white and brown adipose loss and reduces body weight in juvenile mice. Eur J Pharmacol Nov.

[23] Liu Y, Wang J, Zhou X, Cao H, Zhang X, Huang K, Li X, Yang G, Shi X (2020). Mir-324-5p inhibits C2C12 cell differentiation and promotes intramuscular lipid deposition through lncDUM and PM20D1. Mol Ther Nucleic Acids Dec.

[24] Dana R, Bannay A, Bourst P, Ziegler C, Losser MR, Gibot S, Levy B, Audibert G, Ziegler O (2021). Obesity and mortality in critically ill COVID-19 patients with respiratory failure. Int J Obes (Lond) Sep.

[25] Karampela I, Chrysanthopoulou E, Christodoulatos GS, Dalamaga M (2020). Is there an obesity Paradox in critical illness? Epidemiologic and metabolic considerations. Curr Obes Rep Sep.

[26] Noronha NY, Barato M, Sae-Lee C, Pinhel MAS, Watanabe LM, Pereira VAB, Rodrigues GDS, Morais DA, de Sousa WT, Souza VCO, Plaça JR, Salgado W, Barbosa F, Plösch T, Nonino CB (2022). Novel zinc-related differentially methylated regions in leukocytes of women with and without obesity. Front Nutr.

[27] Snipelisky D, Jentzer J, Batal O, Dardari Z, Mathier M (2018). Serum albumin concentration as an independent prognostic indicator in patients with pulmonary arterial hypertension. Clin Cardiol Jun.

