# Supplementary material for: Serum PM20D1 levels in patients with idiopathic pulmonary arterial hypertension and its clinical significance
Source: BMC Cardiovasc Disord. 2024 Apr 13;24:207. doi: 10.1186/s12872-024-03855-6 (PMC11015596; doi:10.1186/s12872-024-03855-6)
Supplement: Supplementary file 1 — Supplementary Material 1 [file 12872_2024_3855_MOESM1_ESM.docx]

**Supporting information of Serum PM20D1 levels in patients with Idiopathic Pulmonary Arterial Hypertension and its clinical significance**

**Results of normal distribution test**

| **normal distribution test** | | | | | | | |
| --- | --- | --- | --- | --- | --- | --- | --- |
|  | Group | Kolmogorov-Smirnov^a^ | | | Shapiro-Wilk | | |
|  |  | statistic | df | Sig. | statistic | df | Sig. |
| Age | 1.00 | .143 | 31 | .108 | .920 | 31 | .023 |
|  | 2.00 | .091 | 72 | .200^*^ | .939 | 72 | .002 |
| BMI | 1.00 | .113 | 31 | .200^*^ | .917 | 31 | .020 |
|  | 2.00 | .107 | 72 | .039 | .955 | 72 | .012 |
| MWD | 1.00 | .107 | 31 | .200^*^ | .955 | 31 | .209 |
|  | 2.00 | .077 | 72 | .200^*^ | .974 | 72 | .134 |
| TC | 1.00 | .110 | 31 | .200^*^ | .949 | 31 | .144 |
|  | 2.00 | .084 | 72 | .200^*^ | .958 | 72 | .016 |
| TG | 1.00 | .123 | 31 | .200^*^ | .949 | 31 | .143 |
|  | 2.00 | .109 | 72 | .034 | .940 | 72 | .002 |
| HDLC | 1.00 | .082 | 31 | .200^*^ | .986 | 31 | .945 |
|  | 2.00 | .079 | 72 | .200^*^ | .961 | 72 | .024 |
| LDLC | 1.00 | .117 | 31 | .200^*^ | .934 | 31 | .055 |
|  | 2.00 | .093 | 72 | .199 | .959 | 72 | .020 |
| ALB | 1.00 | .128 | 31 | .200^*^ | .931 | 31 | .046 |
|  | 2.00 | .110 | 72 | .030 | .940 | 72 | .002 |
| NT | 1.00 | .127 | 31 | .200^*^ | .974 | 31 | .635 |
|  | 2.00 | .080 | 72 | .200^*^ | .952 | 72 | .008 |
| CTNT | 1.00 | .099 | 31 | .200^*^ | .973 | 31 | .594 |
|  | 2.00 | .076 | 72 | .200^*^ | .960 | 72 | .022 |
| PM20D1 | 1.00 | .085 | 31 | .200^*^ | .965 | 31 | .402 |
|  | 2.00 | .089 | 72 | .200^*^ | .940 | 72 | .002 |
| RA | 1.00 | .106 | 31 | .200^*^ | .928 | 31 | .039 |
|  | 2.00 | .107 | 72 | .042 | .971 | 72 | .089 |
| RV | 1.00 | .087 | 31 | .200^*^ | .958 | 31 | .262 |
|  | 2.00 | .101 | 72 | .064 | .964 | 72 | .036 |
| PAW | 1.00 | .096 | 31 | .200^*^ | .956 | 31 | .225 |
|  | 2.00 | .096 | 72 | .098 | .958 | 72 | .016 |
| PAV | 1.00 | .105 | 31 | .200^*^ | .959 | 31 | .277 |
|  | 2.00 | .079 | 72 | .200^*^ | .967 | 72 | .057 |
| TASPE | 1.00 | .103 | 31 | .200^*^ | .961 | 31 | .312 |
|  | 2.00 | .067 | 72 | .200^*^ | .971 | 72 | .097 |
| TASPV | 1.00 | .081 | 31 | .200^*^ | .958 | 31 | .263 |
|  | 2.00 | .119 | 72 | .013 | .927 | 72 | .000 |
| PASP | 1.00 | .139 | 31 | .132 | .959 | 31 | .266 |
|  | 2.00 | .069 | 72 | .200^*^ | .968 | 72 | .063 |
| MPAP | 1.00 | .088 | 31 | .200^*^ | .974 | 31 | .640 |
|  | 2.00 | .070 | 72 | .200^*^ | .977 | 72 | .213 |
| PCWP | 1.00 | .123 | 31 | .200^*^ | .965 | 31 | .388 |
|  | 2.00 | .105 | 72 | .047 | .941 | 72 | .002 |
| PVR | 1.00 | .128 | 31 | .200^*^ | .937 | 31 | .068 |
|  | 2.00 | .093 | 72 | .200^*^ | .950 | 72 | .007 |
| PVRI | 1.00 | .123 | 31 | .200^*^ | .951 | 31 | .165 |
|  | 2.00 | .074 | 72 | .200^*^ | .960 | 72 | .022 |
| MRAP | 1.00 | .091 | 31 | .200^*^ | .959 | 31 | .274 |
|  | 2.00 | .067 | 72 | .200^*^ | .977 | 72 | .221 |

**Results of Student’s t-test in Table 1**

| **Student’s t-test** | | | | | | | | | | | |
| --- | --- | --- | --- | --- | --- | --- | --- | --- | --- | --- | --- |
| group | | Levene test for variance equation | | The t-test of the mean equation | | | | | | | |
|  |  | F | Sig. | t | df | Sig. bilateral) | Mean difference | Standard error value | 95% confidence interval of difference | |  |
|  |  |  |  |  |  |  |  |  | Lower limit | upper limit |  |
| Age | Assuming equal variance | 1.598 | .209 | 2.426 | 101 | .017 | 5.89875 | 2.43109 | 1.07611 | 10.72138 |  |
|  | Assuming unequal variances |  |  | 2.605 | 67.486 | .011 | 5.89875 | 2.26422 | 1.37995 | 10.41754 |  |
| MWD | Assuming equal variance | 10.606 | .002 | -11.585 | 101 | .000 | -103.25504 | 8.91291 | -120.93586 | -85.57422 |  |
|  | Assuming unequal variances |  |  | -9.858 | 41.566 | .000 | -103.25504 | 10.47444 | -124.39985 | -82.11023 |  |
| RV | Assuming equal variance | .001 | .974 | -1.754 | 101 | .082 | -1.60401 | .91440 | -3.41793 | .20992 |  |
|  | Assuming unequal variances |  |  | -1.743 | 56.114 | .087 | -1.60401 | .92030 | -3.44751 | .23950 |  |
| PAW | Assuming equal variance | .077 | .783 | .250 | 101 | .803 | .16045 | .64062 | -1.11037 | 1.43127 |  |
|  | Assuming unequal variances |  |  | .252 | 57.484 | .802 | .16045 | .63793 | -1.11675 | 1.43765 |  |
| PAV | Assuming equal variance | .353 | .554 | .802 | 101 | .424 | .01445 | .01802 | -.02129 | .05020 |  |
|  | Assuming unequal variances |  |  | .821 | 60.175 | .415 | .01445 | .01760 | -.02074 | .04965 |  |
| TASPE | Assuming equal variance | .106 | .745 | .804 | 101 | .423 | .45616 | .56757 | -.66975 | 1.58206 |  |
|  | Assuming unequal variances |  |  | .842 | 63.580 | .403 | .45616 | .54158 | -.62591 | 1.53822 |  |
| PASP | Assuming equal variance | .071 | .790 | .958 | 101 | .340 | .99717 | 1.04098 | -1.06785 | 3.06219 |  |
|  | Assuming unequal variances |  |  | .990 | 61.485 | .326 | .99717 | 1.00721 | -1.01656 | 3.01090 |  |
| MPAP | Assuming equal variance | .127 | .723 | 2.049 | 101 | .043 | 2.31823 | 1.13137 | .07389 | 4.56257 |  |
|  | Assuming unequal variances |  |  | 2.069 | 58.238 | .043 | 2.31823 | 1.12025 | .07600 | 4.56046 |  |
| PVR | Assuming equal variance | .028 | .867 | 6.586 | 101 | .000 | 3.39287 | .51518 | 2.37089 | 4.41485 |  |
|  | Assuming unequal variances |  |  | 6.510 | 55.468 | .000 | 3.39287 | .52119 | 2.34857 | 4.43717 |  |
| PVRI | Assuming equal variance | 5.543 | .020 | -.594 | 101 | .554 | -.05620 | .09466 | -.24399 | .13159 |  |
|  | Assuming unequal variances |  |  | -.656 | 72.649 | .514 | -.05620 | .08561 | -.22684 | .11444 |  |
| MRAP | Assuming equal variance | .712 | .401 | -.373 | 101 | .710 | -.12957 | .34752 | -.81896 | .55982 |  |
|  | Assuming unequal variances |  |  | -.354 | 50.977 | .725 | -.12957 | .36567 | -.86369 | .60456 |  |

**Results of Mann-Whitney test in Table 1**

| **Statistics** | | | | |
| --- | --- | --- | --- | --- |
|  | BMI | RA | TASPV | PCWP |
| Mann-Whitney U | 1112.500 | 428.500 | 351.500 | 495.000 |
| Wilcoxon W | 1608.500 | 3056.500 | 847.500 | 3123.000 |
| Z | -.025 | -4.943 | -5.497 | -4.465 |
| Asymptotic significance (bilateral) | .980 | .000 | .000 | .000 |
|  | | | | |

**Results of ANOVA followed by Tukey’s post hoc test and Bonferroni correction in Figure 1**

| **ANOVA** | | | | | | | | | | |
| --- | --- | --- | --- | --- | --- | --- | --- | --- | --- | --- |
| Dependent variable | | (I) group | | (J) group | | Mean difference (I-J) | Mean difference | Standard error value | 95% confidence interval of difference | |
|  |  |  |  |  |  |  |  |  | Lower limit | upper limit |
| TC | Tukey HSD | dimension2 | 1.00 | dimension3 | 2.00 | -.07503 | .15001 | .871 | -.4292 | .2792 |
|  |  |  |  |  | 3.00 | -.24979 | .14355 | .193 | -.5888 | .0892 |
|  |  |  | 2.00 | dimension3 | 1.00 | .07503 | .15001 | .871 | -.2792 | .4292 |
|  |  |  |  |  | 3.00 | -.17477 | .10793 | .240 | -.4296 | .0801 |
|  |  |  | 3.00 | dimension3 | 1.00 | .24979 | .14355 | .193 | -.0892 | .5888 |
|  |  |  |  |  | 2.00 | .17477 | .10793 | .240 | -.0801 | .4296 |
|  | Bonferroni | dimension2 | 1.00 | dimension3 | 2.00 | -.07503 | .15001 | 1.000 | -.4372 | .2871 |
|  |  |  |  |  | 3.00 | -.24979 | .14355 | .250 | -.5964 | .0968 |
|  |  |  | 2.00 | dimension3 | 1.00 | .07503 | .15001 | 1.000 | -.2871 | .4372 |
|  |  |  |  |  | 3.00 | -.17477 | .10793 | .321 | -.4353 | .0858 |
|  |  |  | 3.00 | dimension3 | 1.00 | .24979 | .14355 | .250 | -.0968 | .5964 |
|  |  |  |  |  | 2.00 | .17477 | .10793 | .321 | -.0858 | .4353 |
| TG | Tukey HSD | dimension2 | 1.00 | dimension3 | 2.00 | -.03072 | .05044 | .815 | -.1498 | .0884 |
|  |  |  |  |  | 3.00 | -.00866 | .04827 | .982 | -.1226 | .1053 |
|  |  |  | 2.00 | dimension3 | 1.00 | .03072 | .05044 | .815 | -.0884 | .1498 |
|  |  |  |  |  | 3.00 | .02206 | .03629 | .816 | -.0636 | .1077 |
|  |  |  | 3.00 | dimension3 | 1.00 | .00866 | .04827 | .982 | -.1053 | .1226 |
|  |  |  |  |  | 2.00 | -.02206 | .03629 | .816 | -.1077 | .0636 |
|  | Bonferroni | dimension2 | 1.00 | dimension3 | 2.00 | -.03072 | .05044 | 1.000 | -.1525 | .0911 |
|  |  |  |  |  | 3.00 | -.00866 | .04827 | 1.000 | -.1252 | .1079 |
|  |  |  | 2.00 | dimension3 | 1.00 | .03072 | .05044 | 1.000 | -.0911 | .1525 |
|  |  |  |  |  | 3.00 | .02206 | .03629 | 1.000 | -.0656 | .1097 |
|  |  |  | 3.00 | dimension3 | 1.00 | .00866 | .04827 | 1.000 | -.1079 | .1252 |
|  |  |  |  |  | 2.00 | -.02206 | .03629 | 1.000 | -.1097 | .0656 |
| HDLC | Tukey HSD | dimension2 | 1.00 | dimension3 | 2.00 | .00706 | .02487 | .957 | -.0517 | .0658 |
|  |  |  |  |  | 3.00 | .00941 | .02380 | .918 | -.0468 | .0656 |
|  |  |  | 2.00 | dimension3 | 1.00 | -.00706 | .02487 | .957 | -.0658 | .0517 |
|  |  |  |  |  | 3.00 | .00235 | .01789 | .991 | -.0399 | .0446 |
|  |  |  | 3.00 | dimension3 | 1.00 | -.00941 | .02380 | .918 | -.0656 | .0468 |
|  |  |  |  |  | 2.00 | -.00235 | .01789 | .991 | -.0446 | .0399 |
|  | Bonferroni | dimension2 | 1.00 | dimension3 | 2.00 | .00706 | .02487 | 1.000 | -.0530 | .0671 |
|  |  |  |  |  | 3.00 | .00941 | .02380 | 1.000 | -.0481 | .0669 |
|  |  |  | 2.00 | dimension3 | 1.00 | -.00706 | .02487 | 1.000 | -.0671 | .0530 |
|  |  |  |  |  | 3.00 | .00235 | .01789 | 1.000 | -.0409 | .0456 |
|  |  |  | 3.00 | dimension3 | 1.00 | -.00941 | .02380 | 1.000 | -.0669 | .0481 |
|  |  |  |  |  | 2.00 | -.00235 | .01789 | 1.000 | -.0456 | .0409 |
| LDLC | Tukey HSD | dimension2 | 1.00 | dimension3 | 2.00 | -.04663 | .08588 | .850 | -.2494 | .1562 |
|  |  |  |  |  | 3.00 | -.42074^*^ | .08218 | .000 | -.6148 | -.2267 |
|  |  |  | 2.00 | dimension3 | 1.00 | .04663 | .08588 | .850 | -.1562 | .2494 |
|  |  |  |  |  | 3.00 | -.37411^*^ | .06179 | .000 | -.5200 | -.2282 |
|  |  |  | 3.00 | dimension3 | 1.00 | .42074^*^ | .08218 | .000 | .2267 | .6148 |
|  |  |  |  |  | 2.00 | .37411^*^ | .06179 | .000 | .2282 | .5200 |
|  | Bonferroni | dimension2 | 1.00 | dimension3 | 2.00 | -.04663 | .08588 | 1.000 | -.2540 | .1607 |
|  |  |  |  |  | 3.00 | -.42074^*^ | .08218 | .000 | -.6191 | -.2223 |
|  |  |  | 2.00 | dimension3 | 1.00 | .04663 | .08588 | 1.000 | -.1607 | .2540 |
|  |  |  |  |  | 3.00 | -.37411^*^ | .06179 | .000 | -.5233 | -.2249 |
|  |  |  | 3.00 | dimension3 | 1.00 | .42074^*^ | .08218 | .000 | .2223 | .6191 |
|  |  |  |  |  | 2.00 | .37411^*^ | .06179 | .000 | .2249 | .5233 |
| ALB | Tukey HSD | dimension2 | 1.00 | dimension3 | 2.00 | -5.50876^*^ | .75636 | .000 | -7.2947 | -3.7228 |
|  |  |  |  |  | 3.00 | -17.24196^*^ | .72379 | .000 | -18.9510 | -15.5329 |
|  |  |  | 2.00 | dimension3 | 1.00 | 5.50876^*^ | .75636 | .000 | 3.7228 | 7.2947 |
|  |  |  |  |  | 3.00 | -11.73321^*^ | .54419 | .000 | -13.0182 | -10.4482 |
|  |  |  | 3.00 | dimension3 | 1.00 | 17.24196^*^ | .72379 | .000 | 15.5329 | 18.9510 |
|  |  |  |  |  | 2.00 | 11.73321^*^ | .54419 | .000 | 10.4482 | 13.0182 |
|  | Bonferroni | dimension2 | 1.00 | dimension3 | 2.00 | -5.50876^*^ | .75636 | .000 | -7.3348 | -3.6827 |
|  |  |  |  |  | 3.00 | -17.24196^*^ | .72379 | .000 | -18.9894 | -15.4945 |
|  |  |  | 2.00 | dimension3 | 1.00 | 5.50876^*^ | .75636 | .000 | 3.6827 | 7.3348 |
|  |  |  |  |  | 3.00 | -11.73321^*^ | .54419 | .000 | -13.0470 | -10.4194 |
|  |  |  | 3.00 | dimension3 | 1.00 | 17.24196^*^ | .72379 | .000 | 15.4945 | 18.9894 |
|  |  |  |  |  | 2.00 | 11.73321^*^ | .54419 | .000 | 10.4194 | 13.0470 |
| NTproBNP | Tukey HSD | dimension2 | 1.00 | dimension3 | 2.00 | 1892.61547^*^ | 129.35663 | .000 | 1587.1662 | 2198.0647 |
|  |  |  |  |  | 3.00 | 3481.91889^*^ | 123.78613 | .000 | 3189.6233 | 3774.2145 |
|  |  |  | 2.00 | dimension3 | 1.00 | -1892.61547^*^ | 129.35663 | .000 | -2198.0647 | -1587.1662 |
|  |  |  |  |  | 3.00 | 1589.30342^*^ | 93.07117 | .000 | 1369.5349 | 1809.0719 |
|  |  |  | 3.00 | dimension3 | 1.00 | -3481.91889^*^ | 123.78613 | .000 | -3774.2145 | -3189.6233 |
|  |  |  |  |  | 2.00 | -1589.30342^*^ | 93.07117 | .000 | -1809.0719 | -1369.5349 |
|  | Bonferroni | dimension2 | 1.00 | dimension3 | 2.00 | 1892.61547^*^ | 129.35663 | .000 | 1580.3117 | 2204.9192 |
|  |  |  |  |  | 3.00 | 3481.91889^*^ | 123.78613 | .000 | 3183.0639 | 3780.7739 |
|  |  |  | 2.00 | dimension3 | 1.00 | -1892.61547^*^ | 129.35663 | .000 | -2204.9192 | -1580.3117 |
|  |  |  |  |  | 3.00 | 1589.30342^*^ | 93.07117 | .000 | 1364.6031 | 1814.0037 |
|  |  |  | 3.00 | dimension3 | 1.00 | -3481.91889^*^ | 123.78613 | .000 | -3780.7739 | -3183.0639 |
|  |  |  |  |  | 2.00 | -1589.30342^*^ | 93.07117 | .000 | -1814.0037 | -1364.6031 |
| CTNT | Tukey HSD | dimension2 | 1.00 | dimension3 | 2.00 | .13300 | .88291 | .988 | -1.9518 | 2.2178 |
|  |  |  |  |  | 3.00 | .19628 | .84489 | .971 | -1.7987 | 2.1913 |
|  |  |  | 2.00 | dimension3 | 1.00 | -.13300 | .88291 | .988 | -2.2178 | 1.9518 |
|  |  |  |  |  | 3.00 | .06328 | .63525 | .995 | -1.4367 | 1.5633 |
|  |  |  | 3.00 | dimension3 | 1.00 | -.19628 | .84489 | .971 | -2.1913 | 1.7987 |
|  |  |  |  |  | 2.00 | -.06328 | .63525 | .995 | -1.5633 | 1.4367 |
|  | Bonferroni | dimension2 | 1.00 | dimension3 | 2.00 | .13300 | .88291 | 1.000 | -1.9986 | 2.2646 |
|  |  |  |  |  | 3.00 | .19628 | .84489 | 1.000 | -1.8435 | 2.2361 |
|  |  |  | 2.00 | dimension3 | 1.00 | -.13300 | .88291 | 1.000 | -2.2646 | 1.9986 |
|  |  |  |  |  | 3.00 | .06328 | .63525 | 1.000 | -1.4704 | 1.5969 |
|  |  |  | 3.00 | dimension3 | 1.00 | -.19628 | .84489 | 1.000 | -2.2361 | 1.8435 |
|  |  |  |  |  | 2.00 | -.06328 | .63525 | 1.000 | -1.5969 | 1.4704 |
| PM20D1 | Tukey HSD | dimension2 | 1.00 | dimension3 | 2.00 | -1.17970^*^ | .12854 | .000 | -1.4832 | -.8762 |
|  |  |  |  |  | 3.00 | -3.13794^*^ | .12301 | .000 | -3.4284 | -2.8475 |
|  |  |  | 2.00 | dimension3 | 1.00 | 1.17970^*^ | .12854 | .000 | .8762 | 1.4832 |
|  |  |  |  |  | 3.00 | -1.95824^*^ | .09248 | .000 | -2.1766 | -1.7399 |
|  |  |  | 3.00 | dimension3 | 1.00 | 3.13794^*^ | .12301 | .000 | 2.8475 | 3.4284 |
|  |  |  |  |  | 2.00 | 1.95824^*^ | .09248 | .000 | 1.7399 | 2.1766 |
|  | Bonferroni | dimension2 | 1.00 | dimension3 | 2.00 | -1.17970^*^ | .12854 | .000 | -1.4900 | -.8694 |
|  |  |  |  |  | 3.00 | -3.13794^*^ | .12301 | .000 | -3.4349 | -2.8410 |
|  |  |  | 2.00 | dimension3 | 1.00 | 1.17970^*^ | .12854 | .000 | .8694 | 1.4900 |
|  |  |  |  |  | 3.00 | -1.95824^*^ | .09248 | .000 | -2.1815 | -1.7350 |
|  |  |  | 3.00 | dimension3 | 1.00 | 3.13794^*^ | .12301 | .000 | 2.8410 | 3.4349 |
|  |  |  |  |  | 2.00 | 1.95824^*^ | .09248 | .000 | 1.7350 | 2.1815 |

**Results of Spearman’s rank correlation analysis in table 2**

|  | | | PM20D1 |
| --- | --- | --- | --- |
| Spearman 的 rho | PM20D1 | correlation coefficient | 1.000 |
|  |  | Sig.（Bilateral） | . |
|  |  | N | 103 |
|  | Age | correlation coefficient | .036 |
|  |  | Sig.（Bilateral） | .721 |
|  |  | N | 103 |
|  | BMI | correlation coefficient | .055 |
|  |  | Sig.（Bilateral） | .578 |
|  |  | N | 103 |
|  | MWD | correlation coefficient | .417^**^ |
|  |  | Sig.（Bilateral） | .000 |
|  |  | N | 103 |
|  | RA | correlation coefficient | -.266^**^ |
|  |  | Sig.（Bilateral） | .007 |
|  |  | N | 103 |
|  | RV | correlation coefficient | -.036 |
|  |  | Sig.（Bilateral） | .720 |
|  |  | N | 103 |
|  | PAW | correlation coefficient | .017 |
|  |  | Sig.（Bilateral） | .868 |
|  |  | N | 103 |
|  | PAV | correlation coefficient | .026 |
|  |  | Sig.（Bilateral） | .798 |
|  |  | N | 103 |
|  | TASPE | correlation coefficient | -.106 |
|  |  | Sig.（Bilateral） | .286 |
|  |  | N | 103 |
|  | TASPV | correlation coefficient | .297^**^ |
|  |  | Sig.（Bilateral） | .002 |
|  |  | N | 103 |
|  | PASP | correlation coefficient | -.089 |
|  |  | Sig.（Bilateral） | .371 |
|  |  | N | 103 |
|  | MPAP | correlation coefficient | -.122 |
|  |  | Sig.（Bilateral） | .220 |
|  |  | N | 103 |
|  | PCWP | correlation coefficient | -.179 |
|  |  | Sig.（Bilateral） | .070 |
|  |  | N | 103 |
|  | PVR | correlation coefficient | -.194^*^ |
|  |  | Sig.（Bilateral） | .049 |
|  |  | N | 103 |
|  | PVRI | correlation coefficient | .113 |
|  |  | Sig.（Bilateral） | .257 |
|  |  | N | 103 |
|  | MRAP | correlation coefficient | .024 |
|  |  | Sig.（Bilateral） | .806 |
|  |  | N | 103 |
|  | TC | correlation coefficient | .098 |
|  |  | Sig.（Bilateral） | .324 |
|  |  | N | 103 |
|  | TG | correlation coefficient | -.017 |
|  |  | Sig.（Bilateral） | .863 |
|  |  | N | 103 |
|  | HDLC | correlation coefficient | -.149 |
|  |  | Sig.（Bilateral） | .134 |
|  |  | N | 103 |
|  | LDLC | correlation coefficient | -.085 |
|  |  | Sig.（Bilateral） | .393 |
|  |  | N | 103 |
|  | ALB | correlation coefficient | .365^**^ |
|  |  | Sig.（Bilateral） | .000 |
|  |  | N | 103 |
|  | NT | correlation coefficient | -.482^**^ |
|  |  | Sig.（Bilateral） | .000 |
|  |  | N | 103 |
|  | CTNT | correlation coefficient | .109 |
|  |  | Sig.（Bilateral） | .273 |
|  |  | N | 103 |

**Results of Logistic regression analysis in table 3**

| **Variables in the equation** | | | | | | | | | |
| --- | --- | --- | --- | --- | --- | --- | --- | --- | --- |
|  | | B | S.E, | Wals | df | Sig. | Exp (B) | 95% confidence interval of difference | |
|  |  |  |  |  |  |  |  | Lower limit | upper limit |
| step 1^a^ | Age | -.029 | .036 | .649 | 1 | .421 | .972 | .906 | 1.042 |
|  | Sex | .825 | .829 | .991 | 1 | .320 | 2.281 | .450 | 11.577 |
|  | BMI | -.159 | .164 | .944 | 1 | .331 | .853 | .619 | 1.176 |
|  | MWD | .053 | .011 | 22.068 | 1 | .000 | 1.055 | 1.031 | 1.078 |
|  | constant | -10.237 | 5.509 | 3.452 | 1 | .063 | .000 |  |  |
|  | | | | | | | | | |

| **Variables in the equation** | | | | | | | | | |
| --- | --- | --- | --- | --- | --- | --- | --- | --- | --- |
|  | | B | S.E, | Wals | df | Sig. | Exp (B) | 95% confidence interval of difference | |
|  |  |  |  |  |  |  |  | Lower limit | upper limit |
| step1^a^ | RA | -.634 | .232 | 7.437 | 1 | .006 | .531 | .337 | .837 |
|  | RV | .393 | .229 | 2.930 | 1 | .087 | 1.481 | .945 | 2.321 |
|  | PAW | .040 | .270 | .022 | 1 | .883 | 1.040 | .613 | 1.765 |
|  | PAV | -9.141 | 8.568 | 1.138 | 1 | .286 | .000 | .000 | 2106.658 |
|  | TASPE | -.271 | .233 | 1.347 | 1 | .246 | .763 | .483 | 1.205 |
|  | TASPV | 2.519 | 1.010 | 6.226 | 1 | .013 | 12.416 | 1.717 | 89.801 |
|  | PASP | .057 | .154 | .137 | 1 | .711 | 1.059 | .783 | 1.431 |
|  | MPAP | -.214 | .220 | .944 | 1 | .331 | .807 | .524 | 1.244 |
|  | PCWP | -1.169 | .609 | 3.686 | 1 | .055 | .311 | .094 | 1.025 |
|  | PVR | -.596 | .272 | 4.809 | 1 | .028 | .551 | .323 | .939 |
|  | PVRI | -1.556 | 1.880 | .685 | 1 | .408 | .211 | .005 | 8.407 |
|  | MRAP | -.727 | .721 | 1.017 | 1 | .313 | .483 | .118 | 1.986 |
|  | constant | 38.523 | 22.771 | 2.862 | 1 | .091 | 5.373E16 |  |  |
|  | | | | | | | | | |

| **Variables in the equation** | | | | | | | | | |
| --- | --- | --- | --- | --- | --- | --- | --- | --- | --- |
|  | | B | S.E, | Wals | df | Sig. | Exp (B) | 95% confidence interval of difference | |
|  |  |  |  |  |  |  |  | Lower limit | upper limit |
| step 1^a^ | TC | 1.161 | .751 | 2.391 | 1 | .122 | 3.193 | .733 | 13.913 |
|  | TG | .004 | 1.760 | .000 | 1 | .998 | 1.004 | .032 | 31.602 |
|  | HDLC | 8.932 | 4.911 | 3.308 | 1 | .069 | 7574.119 | .500 | 1.148E8 |
|  | LDLC | 1.869 | 1.292 | 2.092 | 1 | .148 | 6.483 | .515 | 81.633 |
|  | ALB | 1.146 | .400 | 8.220 | 1 | .004 | 3.145 | 1.437 | 6.882 |
|  | NT | -.002 | .001 | 5.220 | 1 | .022 | .998 | .997 | 1.000 |
|  | CTNT | -.230 | .148 | 2.401 | 1 | .121 | .795 | .595 | 1.063 |
|  | PM20D1 | 3.024 | 1.010 | 8.958 | 1 | .003 | 20.579 | 2.840 | 149.112 |
|  | constant | -53.246 | 18.442 | 8.336 | 1 | .004 | .000 |  |  |
